# Morphometric evaluation of subaxial cervical spine using multi-detector computerized tomography (MD-CT) scan: the consideration for cervical pedicle screws fixation

**DOI:** 10.1186/1471-2474-15-125

**Published:** 2014-04-11

**Authors:** Pongsthorn Chanplakorn, Chaiwat Kraiwattanapong, Kitti Aroonjarattham, Pittavat Leelapattana, Gun Keorochana, Suphaneewan Jaovisidha, Wiwat Wajanavisit

**Affiliations:** 1 Department of Orthopedics, Faculty of Medicine, Ramathibodi Hospital, Mahidol University, 270 Rama VI Road, Phaya Thai, Ratchathewi, Bangkok 10400, Thailand; 2 Department of Orthopedics, Faculty of Medicine, Burapha University, 169 Long-Had Bangsaen Rd. Bangsaen, Muang District, Chonburi 20131, Thailand; 3 Department of Radiology, Faculty of Medicine, Ramathibodi Hospital, Mahidol University, Bangkok, Thailand

**Keywords:** Cervical pedicle, Cervical pedicle screw, Anatomy, Pedicle dimensions, Cervical spine fixation

## Abstract

**Background:**

Cervical pedicle screw (CPS) insertion is a technically demanding procedure. The quantitative understanding of cervical pedicle morphology, especially the narrowest part of cervical pedicle or isthmus, would minimize the risk of catastrophic damage to surrounding neurovascular structures and improve surgical outcome. The aim of this study was to investigate morphology and quantify cortical thickness of the cervical isthmus by using Multi-detector Computerized Tomography (MD-CT) scan.

**Methods:**

The cervical CT scans were performed in 74 patients (37 males and 37 females) with 1-mm slice thickness and then retro-reconstructed into sagittal and coronal planes to measure various cervical parameters as follows: outer pedicle width (OPW), inner pedicle width (IPW), outer pedicle height (OPH), inner pedicle height (IPH), pedicle cortical thickness, pedicle sagittal angle (PSA), and pedicle transverse angle (PTA).

**Results:**

Total numbers of 740 pedicles were measured in this present study. The mean OPW and IPW significantly increased from C3 to C7 while the mean OPH and IPH of those showed non-significant difference between any measured levels. The medial-lateral cortical thickness was significantly smaller than the superior-inferior one. PTA in the upper cervical spine was significantly wider than the lower ones. The PSA changed from upward inclination at upper cervical spine to the downward inclination at lower cervical spine.

**Conclusions:**

This study has demonstrated that cervical vertebra has relatively small and narrow inner pedicle canal with thick outer pedicle cortex and also shows a variable in pedicle width and inconsistent transverse angle. To enhance the safety of CPS insertion, the entry point and trajectories should be determined individually by using preoperative MD-CT scan and the inner pedicle width should be a key parameter to determine the screw dimensions.

## Background

Subaxial cervical spine instability can be caused by various conditions, such as trauma, neoplasm, infection or posterior cervical decompression procedures. In many conditions, the cervical spine stabilization is needed to maintain spinal alignment [1]. Although other surgical techniques such as clamp and hook plating, lateral mass screw fixation or interspinous wiring have been shown effective in stabilizing the cervical spinal column, from the mechanical perspective, the cervical transpedicular screw (CPS) fixation provides a stronger construction than the others and less likely to failure [2-5].

To date, CPS is one of the most advanced procedures for treatment of the cervical instability, and many recent studies have demonstrated the excellent efficacy of its application on the cervical spine surgery [6-10]. Moreover, the advanced intra-operative imaging techniques, such as the navigation-guided spine surgery or three-dimensional image-based navigation systems, can provide a greater accuracy and safety during the CPS insertion which results in the popularity of CPS fixation among cervical spine surgeons [9-12].

However, CPS insertion is a technically demanding procedure, as it carries a risk of catastrophic damage to the surrounding neurovascular structures [8,13-15]. The small size of cervical pedicles and variability in the pedicle morphometry demand a careful assessment of the entry point and the angle of placement of the screws. High percentage of pedicle wall violations has been observed in experimental model [16,17] and even in clinical studies despite the use of intra-operative image guide navigation [9,10]. Therefore, a quantitative understanding of cervical pedicle morphology at different spinal levels would minimize the risk and improve the successful surgical outcome.

Several studies have already been documented regarding the external dimensions and angular parameters of the pedicles [18-23]. To our best knowledge, there are only a few studies documenting the internal architecture of the cervical pedicle, especially the narrowest part of the cervical pedicle or isthmus [15,20,24], which is the crucial part to determine the trajectories and size of the pedicle screw. Therefore, this study aimed to investigate the morphology of the cervical pedicles and quantify the cortical thickness of each cervical pedicle using Multi-detector Computerized Tomography (MD-CT) scan, and to determine the optimal trajectories and size of the cervical pedicle screws.

## Methods

Thai patients who had cervical computerized tomography (CT) imaging at the Department of Radiology, Faculty of Medicine, Ramathibodi Hospital performed for various reasons were recruited and evaluated in this study. The patient was informed about any possibility of participation in the studies involving in the CT imaging prior to perform the CT scan for various conditions and the consent was obtained at the Department of Radiology by the patient, parents or guardians. There were 74 patients in the age group of 18 to 80 years. There were 37 (50%) males with an average age of 54.5 years and 37 (50%) females with an average age of 52.7 years. Patients with an evidence or history of previous cervical spine surgery, infections, neoplasms, trauma or congenital spinal anomalies were excluded from the study. This study was reviewed and had been approved by the Committee on Human Rights Related to Research Involving Human Subjects, Faculty of Medicine Ramathibodi Hospital, Mahidol University [protocol number ID 10-50-42].

**Figure 1:**
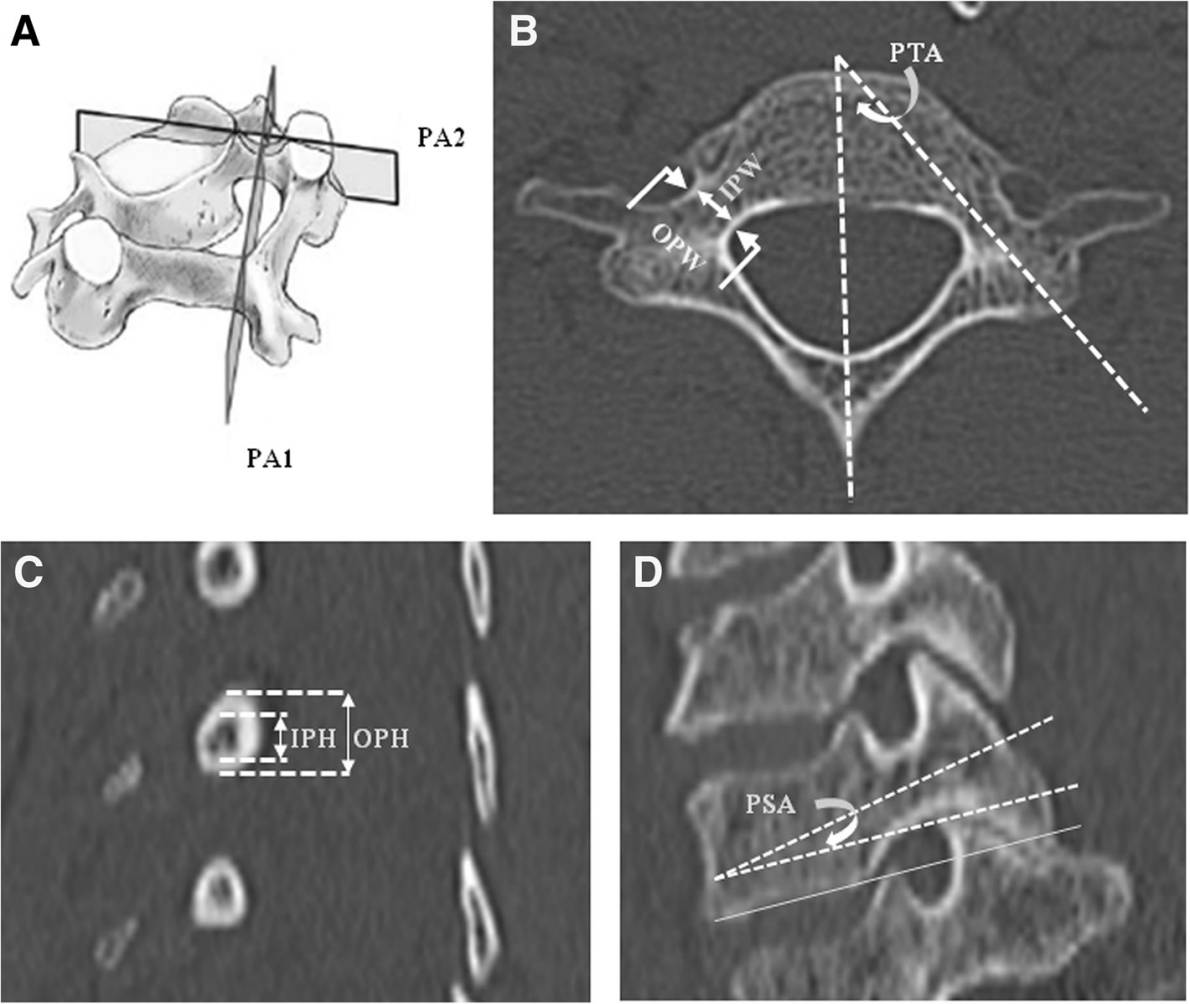
**Illustrated methods used to measure all parameters in the study. A)** Cervical vertebra showing planes used for the Retro-construction image. **B)** Axial reconstruction image through the pedicle isthmus; OPW, IPW and PTA were measured. **C)** Reconstruction image: vertical plane perpendicular to the longitudinal pedicular axis (LPA); PA1 in (a); the OPH and IPH were measured **D)** Reconstruction image: vertical plane through the longitudinal pedicular axis (LPA); PA2 in (a); the PSA was measured. The abbreviations and description are in Table 1.

The cervical CT scans were performed by using a CT scanner (SOMATOM Sensation 64-slice CT scanner, Siemens, Munich, Germany). Axial CT images were obtained with 1-mm slice thickness. Retro-reconstruction into sagittal and coronal planes was then performed to measure various cervical parameters as described by Reinhold et al. [25]. The vertical reconstructions along the plane of longitudinal pedicle axis (LPA) were obtained to measure the pedicle sagittal angle (PSA), the angle between the lower cervical endplate and the longitudinal pedicle axis. Then, the axial reconstructions of the plane perpendicular to the LPA at the pedicle isthmus were employed to measure the outer pedicle height (OPH) and inner pedicle height (IPH). The axial images at the level of pedicle were obtained for the measurement of the outer pedicle width (OPW), inner pedicle width (IPW) and the pedicle transverse angle (PTA), the angle between the sagittal plane and LPA. All of the paired cervical pedicle parameters were measured individually for the left and the right sides using the digital measurement software at the CT work station. The superior-inferior and medial-lateral cortical thickness were obtained from subtraction of the outer parameter with inner parameter along the corresponding axis in each cervical vertebra. The measurements were then calculated as means and standard deviations for each vertebral level. The list of the nomenclature of all parameters that were measured with their abbreviation and description is outlined in Table 1. The measurement method is illustrated in Figure 1.

**Table 1:** Nomenclature for parameters measured on CT reconstruction images

**Measurement**	**Abbreviation**	**Descriptions**
Outer pedicle width	OPW	Outer mediolateral diameter of the pedicle isthmus measured from Axial CT image
Inner pedicle width	IPW	Inner mediolateral diameter of the pedicle isthmus (the width of the cancellous core) measured from Axial CT image
Outer pedicle height	OPH	Outer superoinferior diameter of the pedicle isthmus measured from Axial reconstruction image perpendicular to the longitudinal pedicle axis
Inner pedicle height	IPH	Inner superoinferior diameter of the pedicle isthmus (cancellous core diameter) measured from Axial reconstruction image perpendicular to the longitudinal pedicle axis
Pedicle transverse angle	PTA	The angle between the pedicle axis projection and the anatomical sagittal plane measured from Axial CT image
Pedicle sagittal angle	PSA	The angle between the inferior endplate and longitudinal pedicle axis measured from Axial reconstruction image along the plane of longitudinal pedicle axis

### Statistical analysis

The aforementioned measurements were calculated as means and standard deviations. Unpaired t-test was employed to determine the difference of all dimensional and angular parameters between genders and the left and right pedicles at the same vertebral level. The analysis of variances (ANOVA) with post hoc test was employed to compare all pedicle dimensional and angular parameters among cervical vertebrae of the left and right sides respectively. The post hoc test for linear trend analysis of the individual pedicle parameter at the same side of cervical vertebrae was also performed to verify the stepwise increment among the cervical level. The statistical significance was set for the *p* value less than 0.05. The cortical thickness in height and width dimensions was calculated by subtraction of the outer pedicle diameters by the inner pedicle diameters and analyzed by using the unpaired t-test with 95% confidence interval. All statistical analyses were performed using GraphPad InStat software (version 3.0, GraphPad software, San Diego, CA).

## Results

A total of 740 pedicles from 148 pedicles (74 pair of right and left pedicles) at each cervical vertebra from C3 to C7 were measured in this present study. All measurement parameters were performed at the CT work station by using the measurement software as previously described. The results were divided into 2 parameters as dimensional parameters and angular parameters.

### 1. Dimensional parameters

The mean outer pedicle width (OPW) gradually increased from C3 to C7 as 4.77, 4.86, 5.28, 5.50, and 6.57 mm, respectively, whereas the mean inner pedicle width (IPW) also increased as 2.41, 2.48, 2.78, 3.02, and 3.95 mm, respectively. In contrast, the mean outer pedicle height (OPH) and mean inner pedicle height (IPH) measured from C3 to C7 were relatively constant as demonstrated; the OPH were 5.75, 6.00, 5.79, 5.92 and 6.78 mm, and the IPH were 2.94, 3.11, 3.00, 3.12, and 3.87 mm, respectively (Table 2). There was no significant difference in the dimensional parameters, OPH, IPH, OPW and IPW, between right and left cervical pedicles as illustrated in Table 2. However, the male cervical pedicles had significant larger dimension in comparison to the female pedicles (*p* < 0.05, data not shown), except for the right C6 OPW, left C6 OPH, right C4 and C5 IPW, left C6 IPW and right C3 and C5 IPH which did not show any statistically significant difference (*p* > 0.05, data not shown).

**Table 2:** Dimensional parameters of the cervical pedicles obtained using MD-CT reconstruction

**Vertebra**	**OPW**			**IPW**			**OPH**			**IPH**		
	**Rt**	**Lt**	***p *** **value**	**Rt**	**Lt**	***p *** **value**	**Rt**	**Lt**	***p *** **value**	**Rt**	**Lt**	***p *** **value**
C3												
Overall	4.81 ± 0.83	4.72 ± 0.95	0.54	2.39 ± 0.63	2.44 ± 0.68	0.64	5.78 ± 0.76	5.72 ± 0.72	0.62	2.98 ± 0.80	2.90 ± 0.79	0.54
Male	5.18 ± 0.81	5.18 ± 0.87		2.67 ± 0.66	2.75 ± 0.68		6.05 ± 0.66	6.05 ± 0.66		3.21 ± 0.75^ns^	3.29 ± 0.81	
Female	4.43 ± 0.68	4.27 ± 0.80		2.10 ± 0.45	2.13 ± 0.53		5.51 ± 0.76	5.40 ± 0.64		2.75 ± 0.79	2.51 ± 0.55	
C4												
Overall	4.85 ± 0.85	4.87 ± 0.89	0.89	2.39 ± 0.59	2.58 ± 0.77	0.09	6.01 ± 0.80	5.98 ± 0.80	0.82	3.18 ± 0.77	3.04 ± 0.74	0.26
Male	5.10 ± 0.87^#^	5.24 ± 0.76		2.56 ± 0.64^ns^	2.89 ± 0.73		6.37 ± 0.72	6.32 ± 0.74		3.43 ± 0.80^#^	3.35 ± 0.75	
Female	4.59 ± 0.76	4.51 ± 0.86		2.21 ± 0.47	2.27 ± 0.69		5.64 ± 0.71	5.64 ± 0.71		2.94 ± 0.66	2.72 ± 0.60	
C5												
Overall	5.28 ± 0.88	5.28 ± 0.94	>0.99	2.72 ± 0.72	2.83 ± 0.77	0.37	5.81 ± 0.71	5.77 ± 0.76	0.74	2.97 ± 0.73	3.02 ± 0.70	0.67
Male	5.64 ± 0.85	5.78 ± 0.82		2.91 ± 0.79^ns^	3.10 ± 0.65^#^		6.16 ± 0.60	6.02 ± 0.64		3.21 ± 0.62^ns^	3.24 ± 0.59^#^	
Female	4.91 ± 0.75	4.78 ± 0.78		2.54 ± 0.60	2.56 ± 0.80		5.45 ± 0.64	5.51 ± 0.80		2.72 ± 0.76	2.81 ± 0.73	
C6												
Overall	5.50 ± 0.98	5.51 ± 0.87	0.94	2.95 ± 0.81	3.08 ± 0.75	0.31	5.90 ± 0.87	5.94 ± 0.80	0.77	3.13 ± 0.86	3.12 ± 0.70	0.93
Male	5.72 ± 1.01^ns^	5.78 ± 0.88^#^		3.16 ± 0.83^#^	3.24 ± 0.79^ns^		6.13 ± 0.88^#^	6.24 ± 0.68^ns^		3.48 ± 0.83	3.40 ± 0.68	
Female	5.27 ± 0.90	5.24 ± 0.79		2.75 ± 0.76	2.91 ± 0.68		5.67 ± 0.81	5.64 ± 0.82		2.78 ± 0.75	2.83 ± 0.60	
C7												
Overall	6.54 ± 0.99	6.60 ± 1.00	0.72	3.82 ± 0.98	4.09 ± 1.03	0.10	6.83 ± 0.82	6.72 ± 0.91	0.44	3.93 ± 0.92	3.82 ± 0.86	0.45
Male	6.91 ± 1.03	7.00 ± 0.97		4.27 ± 0.93	4.51 ± 0.96		7.21 ± 0.71	7.05 ± 0.77		4.37 ± 0.79	4.13 ± 0.78	
Female	6.16 ± 0.79	6.21 ± 0.88		3.37 ± 0.82	3.67 ± 0.94		6.45 ± 0.76	6.40 ± 0.92		3.48 ± 0.83	3.51 ± 0.83	

Data showed by means ± standard deviation (mm); *p* value, demonstrated the statistical difference among right and left pedicles atthe representative level; male is larger pedicle dimension compared to female with *p* < 0.01 in all parameters except, ^#^significant with *p* < 0.05, ^NS^no significant between gender (calculated by Unpaired t test); Rt, right pedicle; Lt, left pedicle.

**Figure 2:**
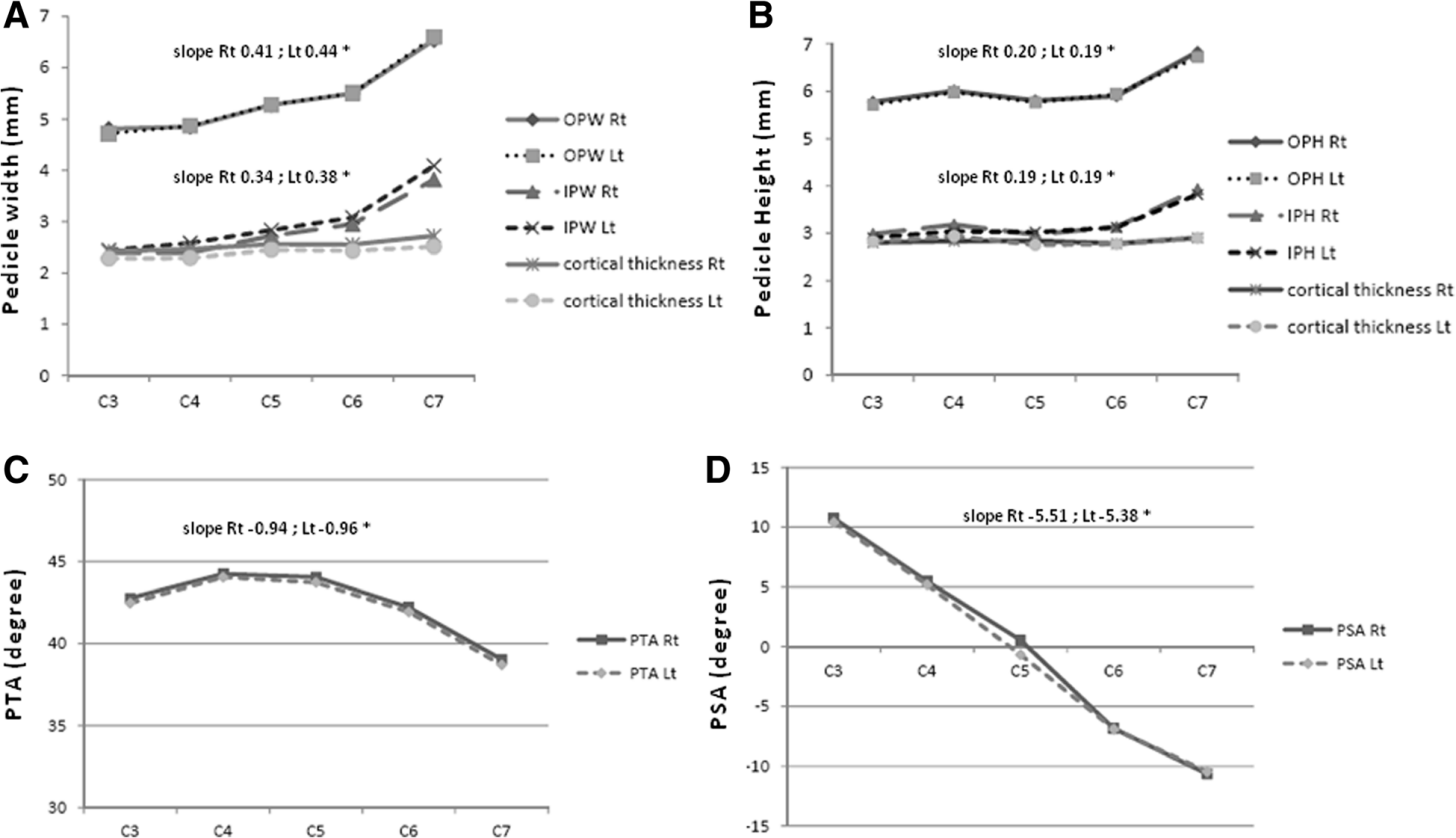
**The means cervical pedicles dimensions and angle in right (Rt) and left (Lt) pedicle. (A)** showed the width dimension and **(B)** showed the height dimension. The cortical thickness of the pedicle in width and height was showed in **(A)** and **(B)** respectively. The PTA was demonstrated in **(C)** and the PSA was demonstrated in **(D)**. *; the slope value of the line below, calculated from ANOVA linear trend statistical analysis.

The dimensional parameters of each pedicle in each cervical vertebra were then analyzed and had demonstrated that the pedicle height parameters, OPH and IPH, did not showed the statistical stepwise difference between the adjacent level (C3 and C4, C4 and C5, C5 and C6), except for C7 that had the largest dimension in both OPH and IPH. The linear trend statistical analysis showed the r squared as 0.123 for right and 0.101 for left OPH and only 0.087 for right and 0.101 for left IPH (Figure 2B). In contrast, the pedicle width dimensions, OPW and IPW, demonstrated the statistically significant stepwise difference between the adjacent levels except for C3-C4 and C5-C6 in both right and left OPW and IPW. However, with the linear trend statistical analysis, the better r squared was demonstrated. For the left pedicle the r squared as 0.299 and 0.292 were shown on OPW and IPW, respectively, and the r squared as 0.279 and 0.270 were found for right OPW and IPW (Figure 2A).

The cervical pedicle cortical thickness is demonstrated in Table 3. The pedicle superior-inferior cortical thickness was greater than that of the medial-lateral cortical thickness. The superior-inferior cortical thickness was quite constant range from 2.75 mm at left C5 pedicle to 2.94 mm at left C4 pedicle. The medial-lateral pedicle cortical thickness ranged from 2.42 at right C3 to 2.72 at left C7 pedicle. Unfortunately, the location of the inner pedicle was not involved in this present study.

**Table 3:** The cortical thickness of cervical pedicles in width and height dimension*

**Vertebra**	**Width dimension**		**Height dimension**	
	**Right**	**Left**	**Right**	**Left**
C3	2.42[2.18-2.66]	2.28[2.01-2.55]	2.80[2.55-3.05]	2.82[2.53-3.07]
C4	2.46[2.22-2.70]	2.29[2.02-2.56]	2.83[2.58-3.08]	2.94[2.69-3.19]
C5	2.56[2.30-2.82]	2.45[2.17-2.73]	2.84[2.61-3.07]	2.75[2.51-2.99]
C6	2.55[2.26-2.84]	2.43[2.17-2.69]	2.78[2.45-3.05]	2.77[2.49-3.05]
C7	2.72[2.40-3.04]	2.51[2.18-2.84]	2.90[2.62-3.18]	2.90[2.61-3.19]

Data showed by difference in means [95% confidence interval] in mm.; *calculated by Unpaired t test.

### 2. Angular parameters

The mean pedicle transverse angles (PTA) from C3 to C7 were 42.62, 44.14, 43.89, 42.07 and 38.87 degrees, (Table 4). There was no statistically significant difference between PTA among right and left pedicle in each cervical level (*p* > 0.05). The PTA variations among C3 to C7 demonstrated the same pattern among the left and right pedicles as they were wider in the upper subaxial cervical spine, C3 to C5 and slightly narrow in the lower cervical region at C6 and C7, as the linear trend statistic showed the slope as −0.94 with r squared 0.1368 on the right pedicles and slope as −0.96 with r squared 0.1422 on the left pedicles (Figure 2C). However, no statistical difference was demonstrated between C4 and C5 PTA but slight statistical differences were found between C3 and C4 PTA, C5 and C6 PTA, respectively (*p* < 0.01). On the contrary, the C6 PTA was significantly wider than C7 PTA (*p* < 0.001). According to gender, the female PTA was slightly wider than male PTA but did not demonstrate a statistically significant difference. However, we found that female PTA demonstrated significantly wider than male on the C6 on the left pedicle and the C5 on both left and right pedicles (*p* = 0.04, 0.02 and 0.007 respectively, data not shown).

**Table 4:** Angular parameters of the cervical pedicles obtained using MD-CT reconstruction

	**PTA**			**PSA**		
**Vertebra**	**Rt**	**Lt**	***p *** **value**	**Rt**	**Lt**	***p *** **value**
C3						
Overall	42.77 ± 3.20	42.47 ± 3.10	0.56	up 10.74 ± 3.01	up 10.40 ± 3.31	0.51
Male	42.21 ± 2.86	42.02 ± 3.14		up 10.62 ± 2.84	up 10.18 ± 3.30	
Female	43.32 ± 3.46	42.91 + 3.04		up 10.86 + 3.21	up 10.62 ± 3.26	
C4						
Overall	44.24 ± 3.56	44.04 ± 3.59	0.73	up 5.47 ± 3.08	up 5.18 ± 3.18	0.57
Male	43.56 ± 3.54	43.48 ± 3.37		up 5.18 ± 2.72	up 5.10 ± 3.22	
Female	44.91 ± 3.49	44.59 ± 3.75		up 5.75 ± 3.41	up 5.27 ± 3.18	
C5						
Overall	44.05 ± 3.27	43.72 ± 3.27	0.54	up 0.56 ± 2.80	dn 0.06 ± 2.57	0.16
Male	43.05 ± 2.51	42.86 ± 2.81		up 0.29 ± 2.85	dn 0.54 ± 2.67	
Female	45.05 + 3.65*	44.59 ± 3.51*		up 0.83 ± 2.76	up 0.40 ± 2.42	
C6						
Overall	42.21 ± 2.63	41.93 ± 2.46	0.51	dn 6.86 ± 3.59	dn 6.91 ± 3.81	0.94
Male	41.54 ± 2.43	41.35 ± 2.44		dn 6.70 + 3.51	dn 6.97 ± 3.69	
Female	42.89 ± 2.68	42.51 ± 2.37*		dn 7.02 ± 3.72	dn 6.86 ± 3.98	
C7						
Overall	39.04 ± 2.90	38.70 ± 2.97	0.48	dn 10.64 ± 3.86	dn 10.45 ± 3.95	0.77
Male	38.62 ± 2.80	38.27 ± 2.70		dn 10.78 ± 3.40	dn 10.81 ± 3.58	
Female	39.45 ± 2.98	39.13 ± 3.19		dn 10.51 ± 4.31	dn 10.10 ± 4.31	

Data showed by means ± standard deviation (degree); *p* value, demonstrated the statistical difference among right and left pedicles at the representative level; gender difference is not demonstrated in all parameters except, *female significant higher than male with *p* < 0.05, (calculated by Unpaired t test); up, upward direction; dn, downward direction; Rt, right pedicle; Lt, left pedicle.

Regarding the pedicle sagittal angle (PSA), the means PSA from C3 to C7 were −10.57, −0.57, −5.33, −0.25, 6.89 and 10.55 degree, respectively (Table 4). There was also no statistically difference between PSA of right and left pedicle in each cervical level (*p* > 0.05). The PSA among the right and the left pedicles also demonstrated the same results as they gradually changed from upward inclination at the upper subaxial cervical spine, C3 to C5, to the downward inclination at the lower cervical region, C6 and C7, with stepwise statistically significant (*p* < 0.001). The linear trend statistics showed slope as 5.509 with r squared 0.845 on the right pedicles and slope as 5.379 with r squared 0.831 on the left pedicles (Figure 2D). Regarding the gender, the results showed no statistically significant difference among the male and female PSA, neither on the cervical levels nor pedicle sides (*p* > 0.05, data not shown).

## Discussion

Of the numerous techniques for stabilizing the cervical spine, transpedicular screw fixation provides the greatest stability. However, the method of fixation is still technically demanding as its carries risk of catastrophic damages to the surrounding neurovascular structures [8,13-15]. To avoid these potential complications, the detailed knowledge of the cervical pedicle anatomy and its architecture as well as the proper surgical techniques and implant design are essential. Recently, Chazono et al. reviewed the ethnic difference in pedicle and bony spinal dimensions but the significant ethnic disparity in pedicle dimension has not been identified [26]. However, from surgical point of view, even minute difference in cervical pedicle dimensions is very crucial, because the small size of the pedicle may not match with the relatively large screw and may result in the pedicle wall violation which has been mentioned in many studies [9,10,16,17].

Our measurement of the pedicle dimension in this present study revealed the characteristic trend, comparable to the previous studies in Asian population [22,26,27]. In this study, the C7 pedicle has the maximal outer pedicle width (OPW) and inner pedicle width (IPW). The means OPW and IPW have demonstrated the statistically significant stepwise difference between the adjacent levels, respectively. On the other hand, the means outer pedicle height (OPH) and means inner pedicle height (IPH) measured from C3 to C7 are relatively constant (Figure 2A and B). However, based on our findings, the height dimensions, OPH and IPH, are larger than the width dimensions, OPW and IPW. Therefore, the precise surgical planning for the proper pedicle screw dimension should be meticulously selected by using the pedicle width on each cervical level to prevent an error due to this morphologic variation.

**Figure 3:**
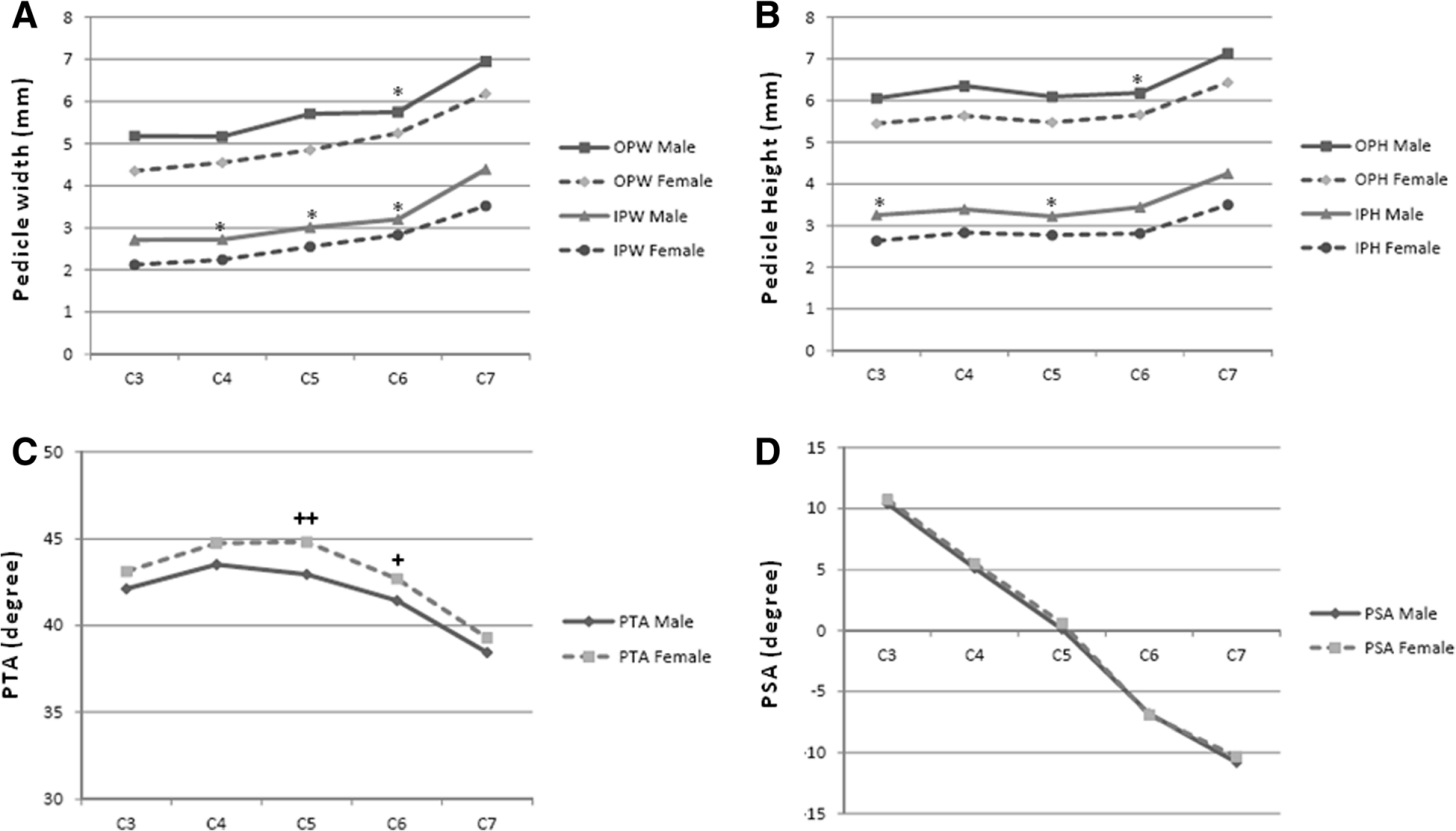
**The means cervical pedicles dimensions and angle in male and female pedicle. (A)** showed the width dimension and **(B)** showed the height dimension. *; indicated no statistical difference between gender (*p* > 0.05) at the left or right pedicle, calculated from ANOVA. **(C)** showed the PTA and **(D)** showed the PSA. +; indicated significant difference between gender at the left pedicle, ++; indicated significant difference between gender at both pedicles, calculated from ANOVA.

The difference between right and left sides in all cervical pedicle dimensions are not demonstrated in this study. In addition, the male cervical pedicles showed significant larger dimensions compared to the female pedicles which is in agreement with the previous studies [23,26,27] but in contrast to the study of Yusof et al. [22] that could not show the gender difference of the cervical pedicles (Figure 3A and B). Ruofu et al. [27] found that 3.5-mm pedicle screw could not be inserted at all cervical levels because of the relatively small pedicle size. The appropriate pedicle diameter for 3.5-mm screw is at least 4.5 mm to allow 0.5 mm bony bridge medially and laterally to avoid pedicle violation. In this present study, we also found that the C3 female pedicles had OPW less than 4.5 mm and might not be suitable for 3.5-mm pedicle screw insertion (Table 2).

Regarding the cortical thickness, the superior-inferior cortical thickness was greater than the medial-lateral cortical thickness. The superior-inferior cortical thickness consistently ranged from 2.75 mm at left C5 pedicle to 2.94 mm at left C4 pedicle. The medial-lateral pedicle cortical thickness ranged from 2.42 at right C3 to 2.72 at left C7 pedicle (Figure 2A and B). These indicated that the cortical shell of cervical pedicle is very thick for at least 1.2 mm in medial-lateral dimension and 1.5 mm in superior-inferior dimension but the inner canal (IPW) is relatively small especially in upper subaxial spine, C3 to C5. These results were comparable to the previous studies [22,28]. Therefore, the small pedicle probe or drill with a diameter of less than 2.5 mm should be suitable to penetrate the pedicle tract, and afterward, the larger probe drill or dilator is then applied until the proper tract diameter for screw insertion is achieved. Unfortunately, the difference of the cortical thickness between superior and inferior and also medial and lateral of the individual pedicle was not evaluated in this study because of the less reliability of measurement after image reconstruction. However, Albumi et al. [7], Panjabi et al. [24] and Gupta et al. [28] pointed out that the medial pedicle cortex is generally thicker than the lateral cortex. Therefore, the guide probe should be in the direction toward the medial pedicle cortex to ensure the safe placement of the pedicle screw.

Concerning the stability of the pedicle screw, Hirano et al. [29] reported that the stability of the pedicle screw is dependent on the thread of the screw engaged in the subcortical bone. In relation to this study, with respect to the engaging screw thread in the cortical shell without breaking the pedicle wall and the screw bone interface, we consider that the self tapping screw with taper configuration and small thread diameter might be more appropriate for the cervical pedicle screw than the current 3.5 mm cortical screw design.

According to the angular parameters, we did not find the statistically significant difference among right and left cervical pedicles of each cervical level in both pedicle transverse angle (PTA) and the pedicle sagittal angle (PSA) (Figure 2C and D). The variation in case of both PTA and PSA among the gender has not been demonstrated in this present study (Figure 3C and D). However, we found that the PTA variation among C3 to C7 demonstrated the same pattern among the left and right pedicles as they had wide angle in the upper subaxial cervical spine, C3 to C5, and became slightly narrow in the lower cervical region at C6 and C7 (Figure 2C). Our results revealed the characteristic trend, which were comparable to the previous studies [17,23,26,27,30]. The PSA among the right and left pedicles also demonstrated the same results as they gradually changed from upward inclination at the upper subaxial cervical spine, C3 and C4, to neutral at the C5 and downward inclination at the lower cervical region, C6 and C7 (Figure 2D). This finding was also similar to the previous studies [17,26,27,30]. However, in our measurement, the C7 PSA showed a significantly larger angle when compared to the C6 PSA. This finding showed a deviation when compared to the previous reports mentioned above in which it had demonstrated the similar PSA between C6 and C7 in most studies. We assume that this result may be caused by measurement error representing the variation in pedicular axis drawing due to the relatively large dimension of the C7 internal pedicle height (IPH) and the variation among the shape of C7 vertebral endplate which may be distorted in a step of image reconstruction.

The ideal entry points and trajectories for cervical pedicle screw insertion has not yet achieved a consensus. Albumi et al. [7] described that the screw entry point should be “slightly lateral” to the center of the articular mass and “closed” to the inferior articular process of the superior vertebra. Karaikovic et al. [31] stated that the entry point was better localized with the use of lateral vertebral notch but did not point out the exact dimension or offset. These descriptions of entry points and trajectories are difficult to follow. Rao et al. [30] also described the surface landmark for the screw entry point by using the lateral margin of lateral mass and also stated that the entry point for each cervical level is variable in both medial and sagittal offset. Ruofu et al. [27] determined the optimal entry point by using the CT reconstruction and found large individual variation in vertical and horizontal offset in reference to the inferior edge of superior articular facet and lateral edge of the lateral mass. Lee et al. [17] studied the optimal entry point by using multidetector computerized tomography (MD-CT) combined with software simulation program. They still found the large variation in both horizontal and vertical offset in reference to the anatomical landmarks, lateral notch, center of the superior ridge and center of lateral mass. They also pointed out that the ideal pedicle axis could only be achieved by the MD-CT reconstruction image.

From our data in this present study, we did find the large variation of the pedicle angles. In addition, the cervical pedicle had a small narrow inner canal and thick outer cortex. Thus, there is no space for the pedicle screw to diverge. Therefore, the entry point and trajectories for cervical pedicle screw insertion should be determined individually by using pre-operative MD-CT scan as pointed out by Lee et al. [17]. Among the pedicle parameters, the pedicle height parameters are relatively constant and this is in contrast to the pedicle width parameters that showed difference among each cervical vertebra. Therefore, the inner pedicle width should be a key parameter to determine the screw dimensions, trajectories and entry point.

## Conclusion

The findings of the pedicle dimension and the angular parameters in this present study reveal the characteristic trend, which were in comparable to the previous studies and in support of the great variability among individuals. Moreover, this study has demonstrated the relatively smaller and variable pedicle width dimensions compared to the height dimensions. In addition, it has been cleared that the cervical pedicle shows a narrow inner canal and thick outer cortex. Thus, the entry point and trajectories for cervical pedicle screw insertion should be determined individually by using preoperative MD-CT scan, and the inner pedicle width should be a key parameter to determine the screw dimensions, trajectories and entry point. Finally, we consider that the self tapping screw with taper configuration and small thread diameter might be a more appropriate design for the cervical pedicle screws.

## Competing interests

The authors declare that they have no competing interests.

## Authors’ contributions

PC: Main researcher who designed and performed the study, performed the statistical analysis and prepared the manuscript; CK: Senior spine surgeon who advised the study design and methods; KA: Orthopedic surgeon who performed the study; PL: Spine surgeon who assisted in the study design and methods; GK: Spine surgeon who assisted in the study design and methods; SJ: Senior radiologist who involved in the study design and helped in manuscript preparation; WW: Senior spine surgeon and corresponding author who approved the study design and methods and statistical analytic results. All authors read and approved the final manuscript.

## Authors’ information

PC, CK and WW are senior spine surgeons who experienced in cervical spine surgery and interested in posterior cervical spine instrumentation and work in Department of Orthopedics, Faculty of Medicine, Ramathibodi Hospital, Mahidol University.

PL and GK are spine surgeons that interested in spinal surgical anatomy and work in Department of Orthopedics, Faculty of Medicine, Ramathibodi Hospital, Mahidol University.

KA is spine fellowship of Spine unit, Department of Orthopedics, Faculty of Medicine, Ramathibodi Hospital, Mahidol University and now work in Department of Orthopedic, Faculty of Medicine, Burapha University, Chonburi.

SJ is senior musculoskeletal radiologist of the Department of Radiology, Faculty of Medicine, Ramathibodi Hospital, Mahidol University.

## Pre-publication history

The pre-publication history for this paper can be accessed here:

http://www.biomedcentral.com/1471-2474/15/125/prepub
